# Supplementary figures and images for: Characterization of dengue virus 3’UTR RNA binding proteins in mosquitoes reveals that AeStaufen reduces subgenomic flaviviral RNA in saliva
Source: PLoS Pathog. 2022 Sep 19;18(9):e1010427. doi: 10.1371/journal.ppat.1010427 (PMC9531803; doi:10.1371/journal.ppat.1010427)

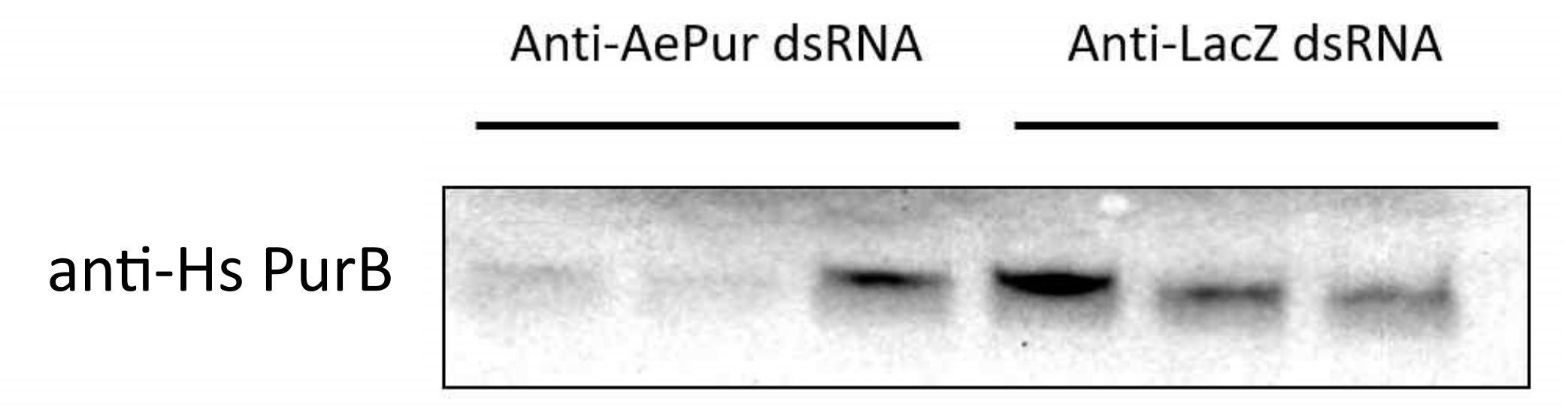

Supplement: S1 Fig — Mosquitoes were injected with either dsRNA against AePur or a dsRNA control against LacZ. Eleven days post-injection, eight mosquitoes were homogenized in RIPA and protein quantity was quantified with microBCA. The same quantity of protein was used for WB and revealed with anti-human PurB. Analysis was done in triplicate and each column represents a replicate. (TIFF) [file ppat.1010427.s001.tiff]

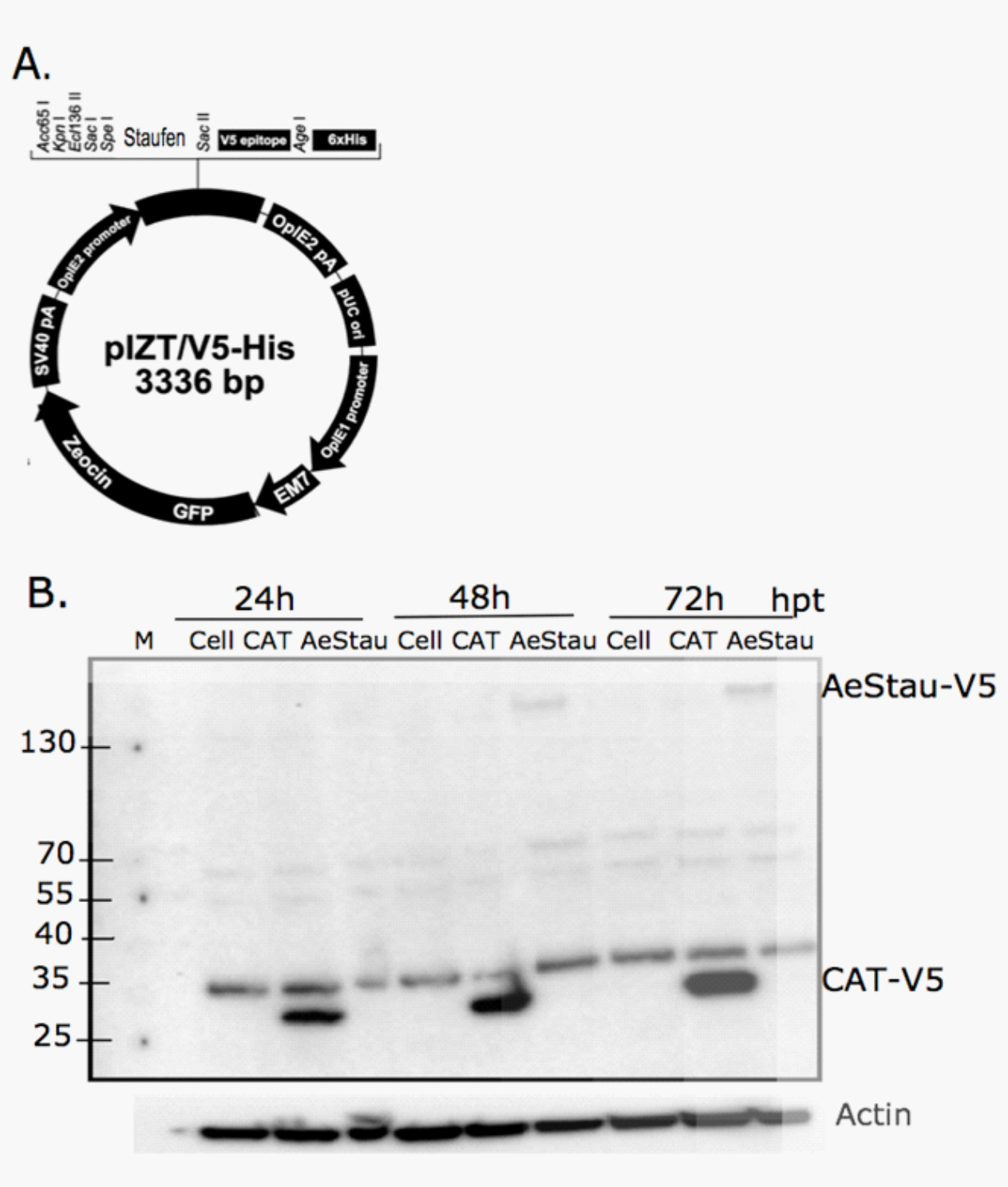

Supplement: S2 Fig — (A) Illustration of the AeStaufen-pIZT/V5 plasmid construct. (B) Western Blot of AeStaufen-V5 (AeStau-V5) at 24, 48 and 72h post-transfection in C6/36 cells. Chloramphenicol Acetyltransferase (CAT)-V5 provided in the kit used to control for expression. Actin was used as loading control. (TIFF) [file ppat.1010427.s002.tiff]

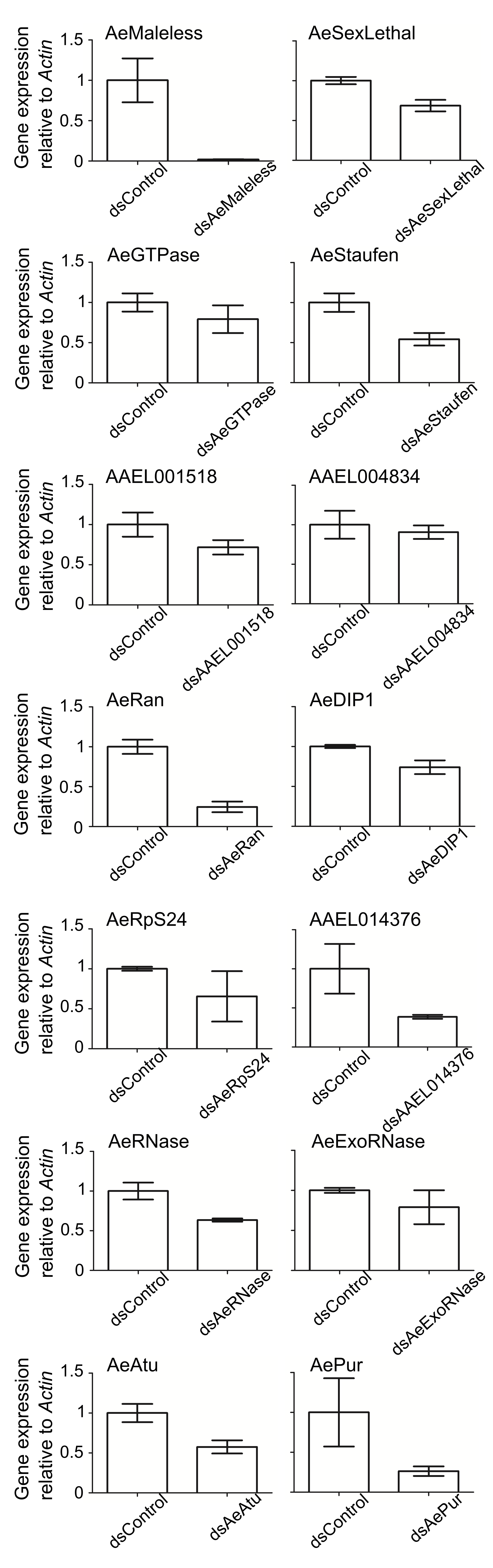

Supplement: S3 Fig — Adult female Ae. aegypti mosquitoes were intrathoracically injected with dsRNA targeting the indicated genes or a dsRNA control targeting LacZ gene. Gene expression was quantified by RT-qPCR four days later in pools of five whole mosquitoes. Data show mean ± s.e.m. from three independent repeats. Reduction in percentage is indicated on the column for the targeted genes. *, p-value < 0.05; ***, p-value < 0.001, as determined by unpaired T-test. (TIFF) [file ppat.1010427.s003.tiff]

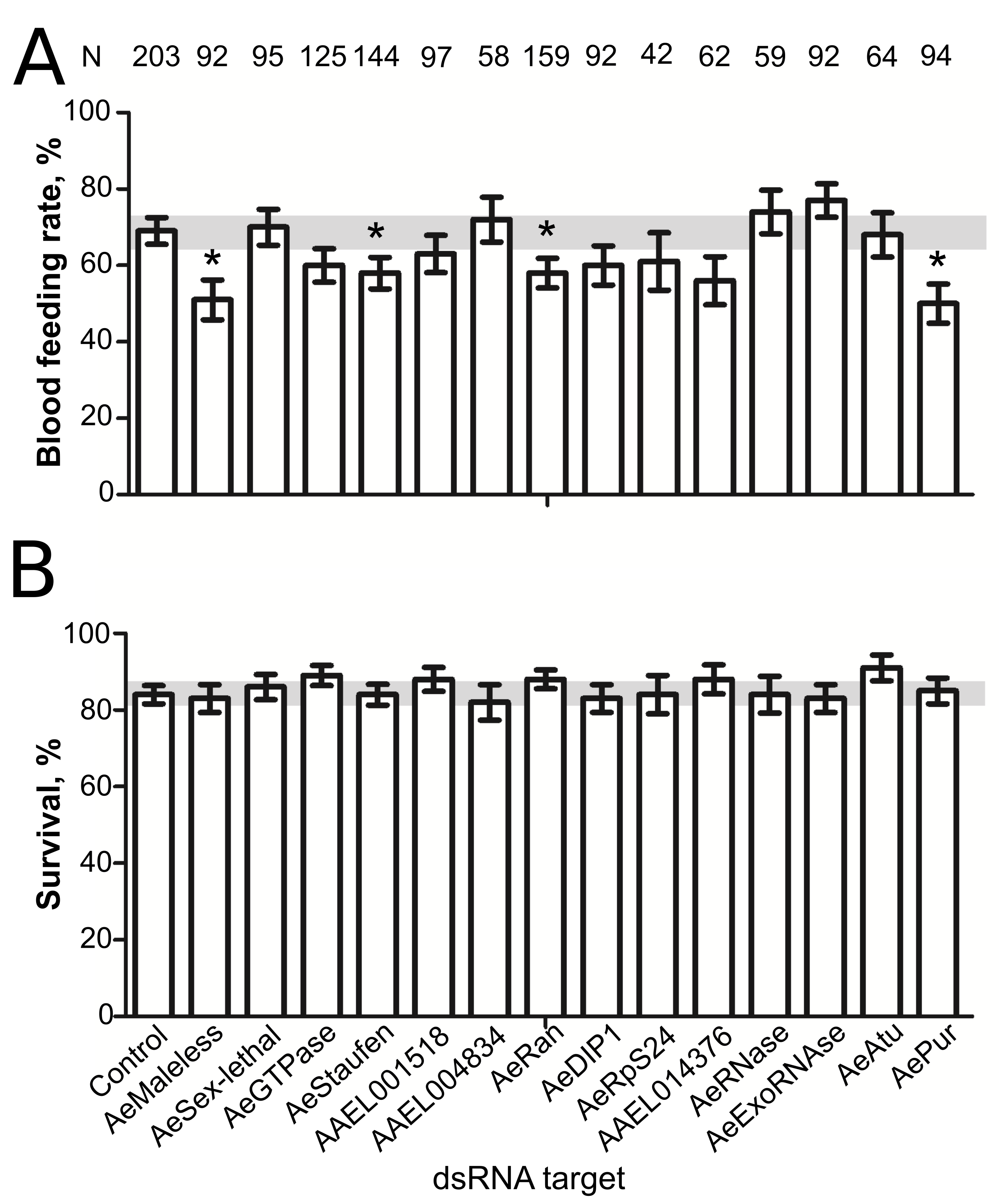

Supplement: S4 Fig — Adult female A. aegypti mosquitoes were intrathoracically injected with dsRNA targeting the indicated genes or a dsRNA control targeting the LacZ gene. Four days post-dsRNA injection, the mosquitoes were exposed to a blood meal containing 106 pfu/ml of DENV. (A) Blood feeding rate. (B) Survival rate determined at 7 days post-feeding. Bars indicate percentage ± s.e. N, number of mosquitoes used for oral feeding in several biological repeats. (TIFF) [file ppat.1010427.s004.tiff]

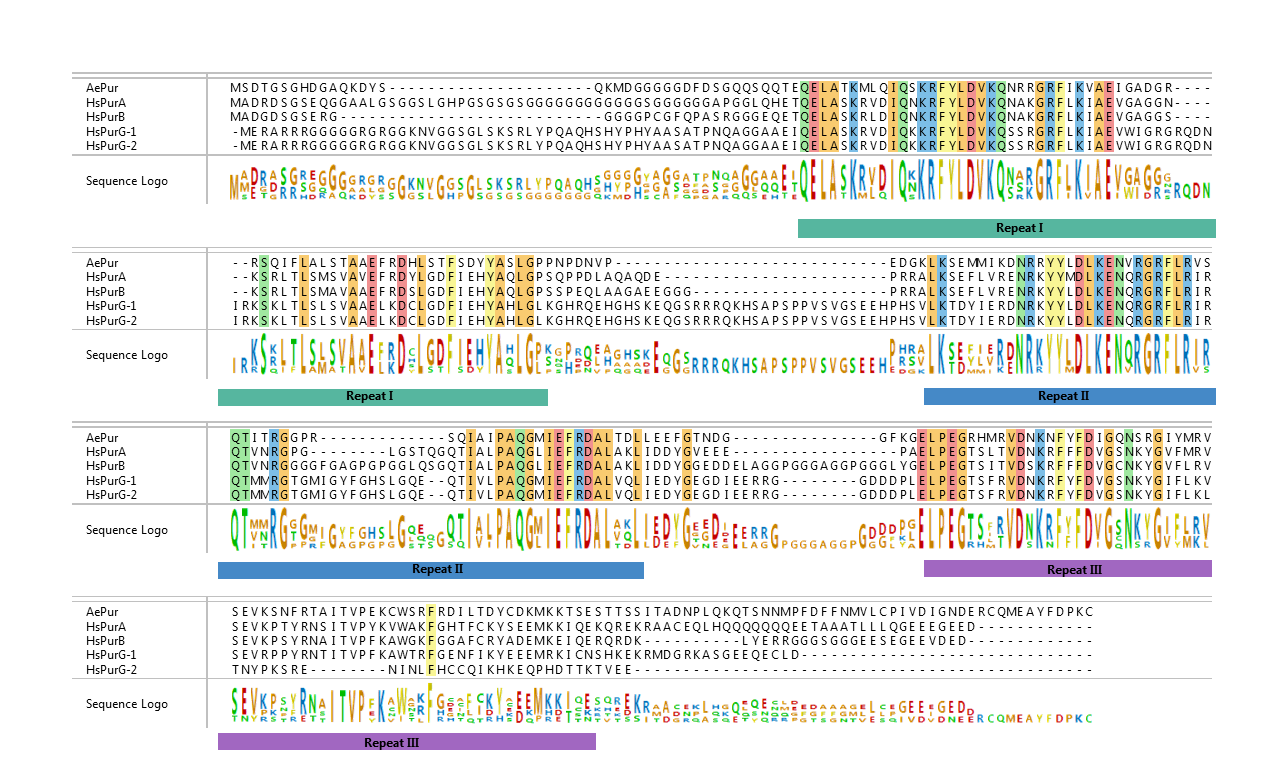

Supplement: S5 Fig — The three repeats of PUR domain are indicated. Conserved amino acids are highlighted. Color coding as follows: yellow, aromatic (F,W, Y); red, acidic (D, E); blue, basic (R, H, K), orange, non-polar (A, G, I, L, M, P, V); and green, polar (C, N, W, S, T). (TIF) [file ppat.1010427.s005.tif]

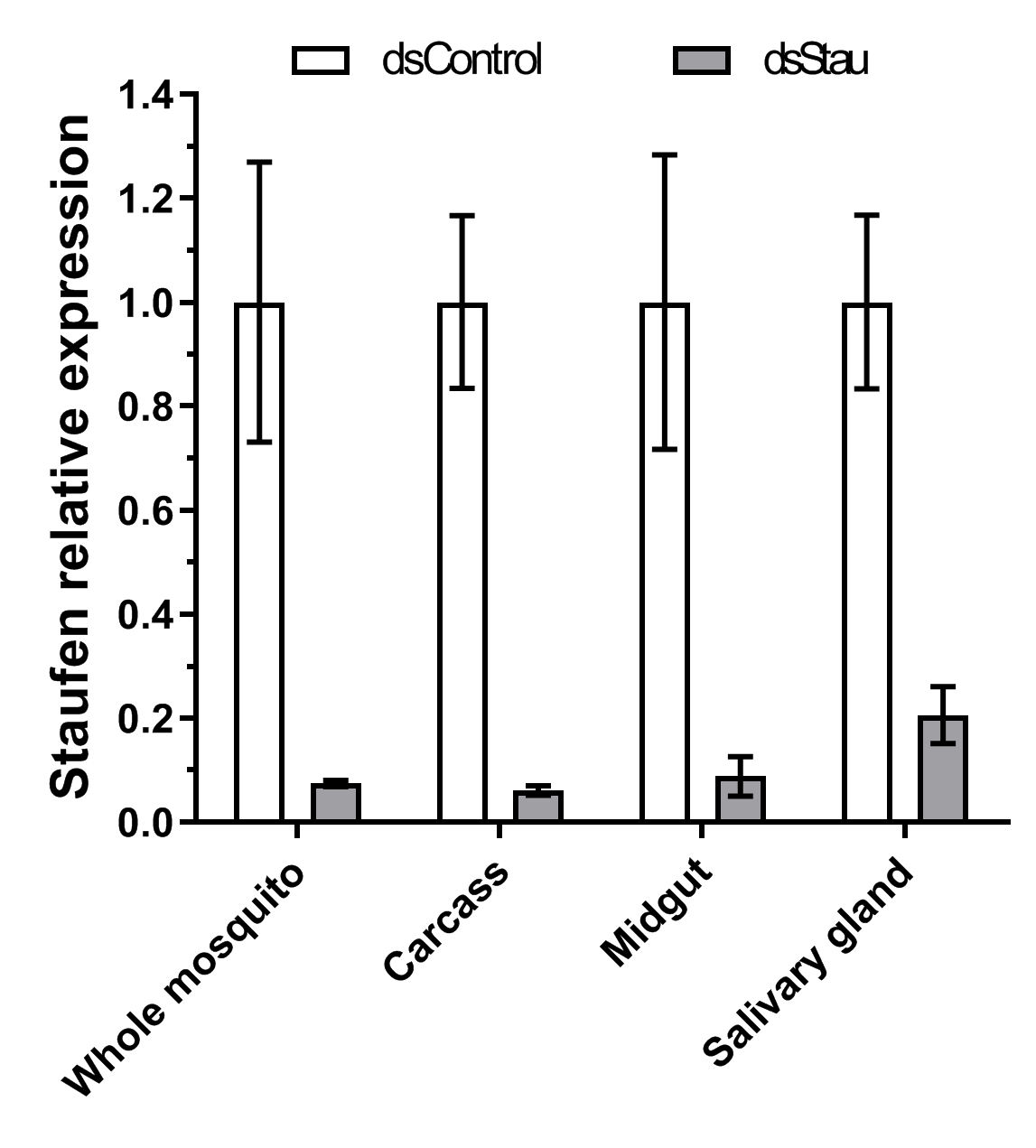

Supplement: S6 Fig — AeStaufen gene expression was quantified at 10 days post-oral infection in pools of ten whole mosquitoes, carcasses, midguts or salivary glands. Actin expression levels were used for normalization. Bars show mean ± s.e.m. from three repeats. (TIF) [file ppat.1010427.s006.tif]

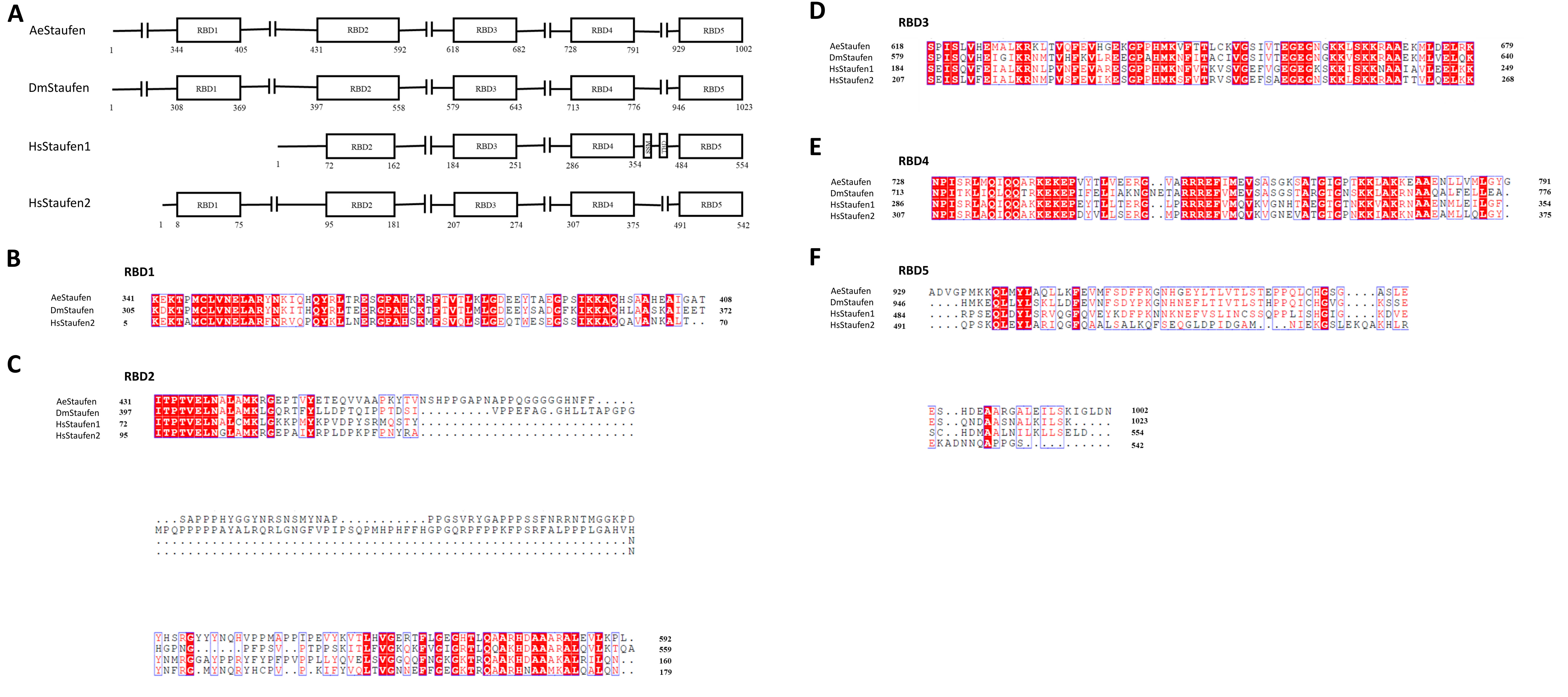

Supplement: S7 Fig — (A) Positions of the RNA-binding domains (RBD) in Staufen homologs. TBD, Tubulin-binding domain. SSM, Staufen-swapping motif. (B-F) Alignment of RBD1-5 in different species. (TIFF) [file ppat.1010427.s007.tiff]
