# Supplementary material for: Characterization of dengue virus 3’UTR RNA binding proteins in mosquitoes reveals that AeStaufen reduces subgenomic flaviviral RNA in saliva
Source: PLoS Pathog. 2022 Sep 19;18(9):e1010427. doi: 10.1371/journal.ppat.1010427 (PMC9531803; doi:10.1371/journal.ppat.1010427)
Supplement: S2 Table — (DOCX) [file ppat.1010427.s009.docx]

# S2 Table. Effect of *AeStaufen* silencing on blood feeding, survival, and salivation rates.

| Date of oral infection | Injected dsRNA | Blood feeding rate^1^, % | Survival rate^2^, % | Salivation rate^3^, % |
| --- | --- | --- | --- | --- |
| 03/08/2018 | dsLacZ | 153/195 = 78 % | 124/153 = 81 % | 45/92 = 48 % |
|  | dsAeStau | 142/190 = 74 % | 114/142 = 80 % | 56/97 = 57 % |
|  | c² p-value | 0.36 | 0.83 | 0.22 |
| 13/07/2018 | dsLacZ | 137/227 = 60 % | 107/137 = 78 % | 87/107 = 80 % |
|  | dsAeStau | 124/196 = 63 % | 100/124 = 80 % | 73/100 = 73 % |
|  |  | 0.53 | 0.69 | 0.24 |
| 01/06/2018 | dsLacZ | 74/131 = 57 % | 51/74 = 68 % | 27/51 = 52 % |
|  | dsAeStau | 64/139 = 46 % | 44/64 = 68 % | 24/44 = 54 % |
|  |  | 0.07 | 1 | 0.85 |

1, Number of engorged mosquitoes / total number of mosquitoes offered a blood meal at 4 days post-dsRNA injection.

2, Number of live mosquitoes / total number of engorged mosquitoes at 10 days post-oral infection.

3, Number of salivating mosquitoes / total number of mosquitoes induced to salivate at 10 days oral infection. Of note, not all live mosquitoes were used for analyzing salivation rates.
