# Supplementary material for: Characterization of dengue virus 3’UTR RNA binding proteins in mosquitoes reveals that AeStaufen reduces subgenomic flaviviral RNA in saliva
Source: PLoS Pathog. 2022 Sep 19;18(9):e1010427. doi: 10.1371/journal.ppat.1010427 (PMC9531803; doi:10.1371/journal.ppat.1010427)
Supplement: S3 Table — (DOCX) [file ppat.1010427.s010.docx]

# S3 Table. Primer pairs used for dsRNA production.

A T7 sequence was added at the 5’ end of each primer.

| Gene name | Gene code | Forward primer | Reverse primer |
| --- | --- | --- | --- |
| *AeMaleless* | AAEL004859 | GATGGGGGTGATTCTGCTC | ACCGACGGCGGTGTATTTAT |
| *AeSex-lethal* | AAEL011150 | CCGATTGTGGGGAATGTC | CTTCGGACAGGAAGTTAACGA |
| *AeGTPase* | AAEL003813 | GTGATAGCACCAAAATTCAGCA | AACCGGTTGGAGCTGAGAAT |
| *AeStaufen* | AAEL007470 | CACCGGGGAGCGTAAGATAC | GTGCACGAGGGAGATTGG |
| *AAEL001518* | AAEL001518 | CGGACGATGAGCAACTTTTC | GGTGCCGTTTACCAGTGTAAT |
| *AAEL004834* | AAEL004834 | AGCAGCTTCAGCAAGCAAAT | CGGATGGATGCCAGTTTG |
| *AeRan* | AAEL009287 | AGTCCGCATTCAACCGTCT | CACGGTTCGTGTGGAACA |
| *AeDIP1* | AAEL012964 | GAAACCAAGACCACCACCAA | GACGCATCAATGCAAGAAGA |
| *AeRpS24* | AAEL014292 | CAAAATGTCGACCGCTACAA | TGCGGGTCATCTTCTTCTTC |
| *AAEL014376* | AAEL014376 | CGTTTCTACAACATGCACGG | GCGATCGGAACGAATGTAGT |
| *AeRNase* | AAEL001089 | CGAAATTGGAGGCCAAAC | TGCCTTATGCACAACGAAAT |
| *AeExoRNase* | AAEL002475 | TTGCATTTGCCAAACTCATC | CGATGCTTGGTATGGTGTTG |
| *AeAtu* | AAEL006172 | ACAAGCTCATCGATTCCGAC | CGTCCAGAGTTTCCTCTTCG |
| *AePur* | AAEL012134 | AAACCGAACAAGAGCTGGC | GACGTTCTCCTTCAGGTCCA |
| *LacZ* | Control | ACACCAACGTGACCTATCCC | CCGCCACATATCCTGATCTT |
