# Supplementary material for: Characterization of dengue virus 3’UTR RNA binding proteins in mosquitoes reveals that AeStaufen reduces subgenomic flaviviral RNA in saliva
Source: PLoS Pathog. 2022 Sep 19;18(9):e1010427. doi: 10.1371/journal.ppat.1010427 (PMC9531803; doi:10.1371/journal.ppat.1010427)
Supplement: S4 Table — (DOCX) [file ppat.1010427.s011.docx]

# S4 Table. Primers used for Real-Time qPCR

| Gene name | Gene code | Forward primer | Reverse primer |
| --- | --- | --- | --- |
| *AeMaleless* | AAEL004859 | TGCAGGTGACTTTCCCGTAT | CCATGTTGTCGACCCATTCC |
| *AeSex-lethal* | AAEL011150 | GTGGAAGGAACGCCTCGGATA | GGAGCGAGTGCGACATTC |
| *AeGTPase* | AAEL003813 | TTCCACACTTCCGCCAACCT | GAAGGATCCAGCCGTGGTCA |
| *AeStaufen* | AAEL007470 | GAAGTGGTGGTGATGGAGAA | TTTGTTTGCAATGCCTGAAT |
| *AAEL001518* | AAEL001518 | CCACAAGCTGCACCGGTTAC | CCTTGGCACGCTTGGAAACA |
| *AAEL004834* | AAEL004834 | TTGCGAGATAGCGCCAAGGA | CTGCATTTGCATCGGCAGGT |
| *AeRan* | AAEL009287 | AAATTGCGATTCCGAGTTTC | CATAAGGTCCGTTTCTGGGT |
| *AeDIP1* | AAEL012964 | ACGATTCTACGATGCCGGTC | TCAGATTCTTGCGGTTGGCA |
| *AeRpS24* | AAEL014292 | TCGAACCCAAGCACCGACT | CCGCGCACCTTCTTCATACG |
| *AAEL014376* | AAEL014376 | CCGGTATGGCAAGGGATTTG | GCTCCGTTAGTTCCTGCTTG |
| *AeRNase* | AAEL001089 | ATTGAAACTCGCCGACGAAC | AAAGATCGTTCCAGGCAACT |
| *AeExoRNase* | AAEL002475 | CAGCACAACTCCGTGAAGGC | CCTCGCTGTTGCCGAAAGAC |
| *AeAtu* | AAEL006172 | GCGCTCTGGAAGGTCTCGAT | GAGCGAGCAGGCTTCCACT |
| *AePur* | AAEL012134 | CCGATTACTGCGACAAGATG | TTTCTCTCTCGCTCTCGTCA |
| *Actin* | AAEL011197 | GAACACCCAGTCCTGCTGACA | TGCGTCATCTTCTCACGGTTAG |
