# Supplementary material for: Characterization of dengue virus 3’UTR RNA binding proteins in mosquitoes reveals that AeStaufen reduces subgenomic flaviviral RNA in saliva
Source: PLoS Pathog. 2022 Sep 19;18(9):e1010427. doi: 10.1371/journal.ppat.1010427 (PMC9531803; doi:10.1371/journal.ppat.1010427)
Supplement: Attachment — Submitted filename: Asnwers to Reviewer comments.docx [file ppat.1010427.s012.docx]

Dear Reviewers and Editors,

We are extremely thankful to all reviewers for pointing out the novelty and quality of our study. We have addressed all comments in a point-by-point answer, presented below. Importantly, we have generated new data to confirm the specificity of the anti-Human Pur antibody against mosquito Pur. Please see our answers in **blue**.

Julien Pompon, PhD.

Reviewer's Responses to Questions

**Part I - Summary**
Please use this section to discuss strengths/weaknesses of study, novelty/significance, general execution and scholarship.

Reviewer #1: The host factors that positively or negatively modulate dengue virus infection in mosquitoes are still poorly characterized. The authors used RNA-affinity chromatography and mass spectrometry to identify RNA-binding proteins interacting with the dengue virus 3’UTR in Aedes aegypti cells, followed by their functional validation in mosquitoes in vivo. This is a well conducted study, and the manuscript is clearly organized and written. The topic is relevant and the approach is innovative. My (mostly minor) suggestions to improve the paper are below.

Reviewer #2: The genome and the replicative forms of Dengue viruses, as is the case with other RNA viruses, is able to interact with RNA binding proteins (RBPs). While the importance of RBPs has been documented in humans, there is poor information about the relevance of these factors in DENV transmission by mosquitoes. In the present manuscript authors identified RBPs which interact with DENV2 3’UTR in Ae. aegypti cells. Authors confirmed the interaction of AePur to the 3’UTR, whereas AeStaufen interacts with both 3’UTR and sfRNA. The proviral function of AeRan, AeExoRNase, and AeRNase was evaluated by in vivo functional assay, whereas AeGTPase, AeAtu, and AePur have anti-viral functions in mosquitoes.
Authors also found that AeStaufen mediates a reduction of gRNA and
sfRNA copies in several mosquito tissues, revealing AeStaufen’s role in DENV transmission.
The manuscript is interesting and relevant, however there are some aspects that should considered to fully support authors conclusions.

Reviewer #3: “Characterization of dengue virus 3’UTR RNA binding proteins in mosquitoes reveals that AeStaufen reduces subgenomic flaviviral RNA in saliva” by Shih-Chia Yeh et al.,

The main findings: DENV shuttle between vertebrate hosts and mosquitoes. Although many human RNA-binding proteins (RBPs) have been identified with important roles during DENV life cycle in human cells, the role of mosquito RBPs in regulating DENV infection cycle is less investigated. Using RNA-affinity chromatography followed by mass spectrometry, the authors report in this paper on the identification of fourteen Aedes aegypti proteins which associate with DENV2 3’UTR. They then choose two candidates to verify these associations. They further conduct in vivo functional analysis of these candidates in mosquito and identify 3 pro-viral candidates (AeRan, AeExoRNase and AeRNase) and 3 anti-viral candidates (AeGTPase, AeAtu and AePur). Interestingly, though human homologs of AePur also associate with DENV2, these human proteins do not have a role in DENV2 infection in the cultured cells. Whereas the initial investigation by RNAi in mosquito does not identify a role of AeStaufen in DENV2 infection, detailed analyses of AeStaufen function in mosquito tissues show that it suppresses both gRNA and sfRNA levels in several tissues including the salivary gland. Of interest, AeStaufen reduces sfRNA:gRNA ratio in the saliva without affecting viral particles.

Novelty/significance: As a single-strained RNA virus, the life cycle of DENV in mosquito is tightly regulated by mosquito factors. Among these factors, RNA binding proteins play important roles in controlling DENV gRNA translation, degradation, replication, and virion assembly. Although similar approach has already being developed to identify Aedes proteins which bind to 3’UTR of Zika virus, this study is the first attempt to identify Aedes proteins associate with DENV2 3’UTR and provides some useful information for follow up study. Overall, the study is well designed and this is an interesting piece of work with novel aspects. It offers some insights on how DENV2 infection and replication in mosquito is regulated via its 3’UTR associated proteins, which warrant further investigation.

Weakness: Some methodology is not well explained. Some texts are a bit confusing and need to be rephrased. Additional data could be considered to support the authors’ claim on the “transmission inhibition” role of AeStaufen. Addressing/clarifying these would strengthen the conclusion present in this manuscript.

**Part II – Major Issues: Key Experiments Required for Acceptance**
Please use this section to detail the key new experiments or modifications of existing experiments that should be absolutely required to validate study conclusions.

Generally, there should be no more than 3 such required experiments or major modifications for a "Major Revision" recommendation. If more than 3 experiments are necessary to validate the study conclusions, then you are encouraged to recommend "Reject".

Reviewer #1: (No Response)

Reviewer #2: Major concerns

1. Although the RNA-affinity chromatography coupled with mass spectrometry (MS) using mosquito cell lysates is an appropriate strategy to analyze RBPs, authors only used uninfected mosquito cell lysates. It is possible that the RBPs identified from uninfected cell lysates may differ from the ones identified in infected cell lysates. The validation of the RBPs should be performed in the presence of infected cell lysates.

The use of infected material for RNA-affinity chromatography is indeed an approach that we discussed while planning for the study. However, we preferred to work with uninfected material to prevent competition between the virus-produced 3’UTR and sfRNA and the “artificial” 3’UTR” introduced for RNA-affinity. Such a competition may have limited the detection of 3’UTR-interacting proteins. Nonetheless, to make sure that the RBPs we identified were relevant in an infection context, we subsequently validated the interactions between RBP and viral RNA using RIP in infected cells and mosquitoes for two RBPs.

We thank the reviewer for pointing to this compromise in our approach and have now clarified this point in:
-the introduction: L. 115. “We validated these findings in infection condition…”
-the results: L.137-141 “Of note, we chose not to use infected Aag2 lysates to prevent competition between the virus-produced 3’UTR and sfRNA and the “artificial” 3’UTR introduced for RNA-affinity. Nonetheless, to confirm the RBP-viral RNA interactions identified by RNA-affinity chromatography, we subsequently used RNA immunoprecipitation (RIP) in infected mosquitoes and cells, as detailed in the following subsection.”

2. How do the authors know that the RBPs identified are bound directly to the RNA? Is it possible that the proteins are not binding directly to the RNA and are part of a complex which is bound to the RNA?

We thank the reviewer for pointing to this alternative hypothesis and have added this in the results section:
L. 157-159: “While most of the interacting RBPs have a RNA binding domain, these and the others could either directly bind to the 3’UTR or indirectly through a complex of proteins.”

3. Have the authors confirmed the interaction of the identified proteins with gRNA of sfRNA by mobility shift assays?

This is an interesting suggestion. However, we did not choose to perform EMSA as this technology does not reproduce the full complexity of the infected milieu. For EMSA, we would need to produce the protein in a heterologous system that may modify the protein structure and then synthetize sfRNA or gRNA. Mixing the two components may then reveal interactions through mobility shift. However, we see several limitations to EMSA: (i) it does not include other proteins which may influence RBP interactions, as suggested above (this reviewer point 2); (ii) we may not reproduce the physiological conditions of the cells, ie pH, salt concentrations… that may influence RBP binding; (iii) RBP interactions with viral RNA may only occur in some cellular compartments and EMSA may reveal interaction by forcing proximity between the protein and the RNA, as with our RNA-affinity chromatography.

Instead, we used RNA immuno-precipitation (RIP) from infected cells and infected mosquitoes to confirm RBP interactions with gRNA and sfRNA. By revealing interactions between RBPs and virally-produced 3’UTR in infected condition, RIP offers information that is supportive and complementary to the RNA-affinity chromatography, for which we used uninfected material.

We now clarified the rationale behind the use of RIP by adding:
L. 170-172: “RIP provided supportive and complementary information to the RNA-affinity chromatography by revealing *in vivo* interactions between proteins and virally-produced 3’UTR.”

4. Have the authors analyzed the location of the identified RBPs in infected cells? Have these proteins the same location than viral RNA?

While interesting, this suggestion is more tedious than it looks in mosquitoes and would require substantial amount of work. First, sfRNA having the same sequence as the genomic 3’UTR, its identification requires a dual FISH labelling. Even with this strategy, the proximity between gRNA and sfRNA may not allow its clear distinction. Second, there are very few antibodies against mosquito proteins. The successful use of an antibody against Human PurB to detect mosquito Pur was more an exception than the rule. Indeed, an antibody against Human Staufen that we tested did not work for the mosquito Staufen. Although the suggestion is interesting, we were not able to conduct this approach. However, to ascertain of the RNA-protein interactions, we have deployed two different approaches (ie RNA-affinity and RIP), which we think can support the interactions.

5. Have the authors confirmed some of the interactions using the 3’UTR of another DENV serotype? Have the authors analyzed the role of AeStaufe in the infection with others DENV serotypes in mosquito?

We have not confirmed the interactions with other DENV1, 3 and 4 3’UTR. However, from our previous experiments in human cells, the 3’UTR-interacting RBPs widely differ between the different serotypes (Liao et al, 2018. RNA). To clarify this point, we have now added this information:

L. 159-160: “As demonstrated previously in human [21], RBPs interacting with the 3’UTR of DENV2 may widely vary from RBPs interacting with the other DENV serotype 3’UTR.”

Reviewer #3: In several places (line 40-41 in Abstract, line 62-65 in Author abstract, line 337-338 and line 431-432 in Discussion), the authors state that “AeStaufen impacts on viral transmission”. However, data present in this manuscript are in line with their claim that “AeStaufen reduces subgenomic flaviviral RNA in saliva” (in the title) but do not lend support on its role on viral transmission. Interestingly, saliva from AeStaufen knockdown mosquito show higher sfRNA:gRNA ratio without affecting viral titers. This provides a unique situation to test whether AeStaufen has an impact on viral transmission (via controlling the sfRNA:gRNA ratio). The authors could use the infection assay established in their ref 28 to infect human cells with saliva collected from control or AeStaufen knockdown mosquitoes. If saliva derived from AeStaufen knockdown is more infectious than saliva from control knockdown, it will not only demonstrate the role of AeStaufen during DENV2 infection cycle but also give strong support to the role of sfRNA during virus infection in general.

We thank the reviewer for this suggestion. Indeed, our claim that AeStaufen influences transmission is based on another paper that is under review. In this other paper, we provide full support to show that sfRNA is secreted inside vesicles in saliva and that higher sfRNA:gRNA ratio increases infection of human skin cells.

As this is the case in Science, we incrementally built knowledge and used the conclusion from the study under review to discuss the implications of our finding for AeStaufen.

**Part III – Minor Issues: Editorial and Data Presentation Modifications**
Please use this section for editorial suggestions as well as relatively minor modifications of existing data that would enhance clarity.

Reviewer #1: Main comments:

In the RNA immunoprecipitation assays (Figure 2), the sfRNA/gRNA ratio in immunoprecipitates could simply reflect their ratio in the protein lysate, not the differential binding affinity of candidate RBPs. This analysis should account for the original ratio, which is presumably different between live mosquitoes and C6/36 cells.

We are sorry for the misunderstanding but the input for either the protein or the control IP was the same and therefore contained the same sfRNA:gRNA ratio. To clarify this point, we added:

L. 178-179: “Variation in enrichment between AePur and IgG IP indicated variation in affinity as we used the same input in both conditions.”

The authors claim that they “validated gene silencing” (line 195) but the data presented in Figure S2 indicate that silencing efficiency was highly variable among target genes. It would be useful to report the % reduction in gene expression level and its statistical significance, as well as discuss the implications of incomplete silencing for some of the candidate genes.

As suggested, we have modified Figure S2 to add the percentage of silencing and the significance. Furthermore, we modified the text to discuss the implications of incomplete silencing:

L. 206-212: “Four days post dsRNA-injection, we quantified gene silencing in whole mosquitoes and observed a wide variation in silencing efficiency (Fig. S3). Variation in mRNA levels should be interpreted with caution as it does not directly correlate with protein levels and could either under- or over-estimate protein depletion. Nonetheless, in absence of antibodies for protein quantification, we considered that a decrease in mRNA associated with a change in phenotype indicated a successful protein depletion.”

There are some statistical issues with the analysis of proportions (infection rate, feeding rate, survival rate) such as those shown in Figure 3A and Figure S3. In Figure 3A, the use of Z test does not seem appropriate to compare infection rates because Z test is only valid for a continuous variable with a large sample size and a normally distributed residual variance. By nature, percentages cannot be normally distributed because they have lower and upper limits (0 and 100). Moreover, calculating the average of percentages is incorrect unless the three experimental repeats had the exact same sample size, or if the average was weighted. In general percentages are more safely analyzed as a binary variable with a 95% confidence interval, which directly accounts for the sample size.

We thank the reviewer for addressing these statistical inaccuracies. We have now modified the stats to use Comparative Error test for percentage comparisons, i.e. in Fig 3A, and S3. The new test revealed a significant effect of AeStaufen and AeRan silencing on blood feeding. This information was updated in the result section.
With respect to combining results from different experiments, we would like to state that we did not combine the percentages but the actual numbers and then calculated the percentage on the pooled data. We have modified the methods to detail these.
L. 689-692: “Percentages for infection, blood-feeding and survival rates were calculated by pooling data from several experiments and by calculating the overall percentages based on the actual mosquito numbers. Standard error for percentages were estimated by calculating the square-root of [p (1-p)/n], where p is the proportion and n the number of observations. Differences in percentages were evaluated with the comparative error test.”

Pooling experimental repeats (for example, Figure 3, Figure S3) could be misleading if the repeats were inconsistent. In general, pooling is only justified when a potential experiment effect (or experiment x treatment interaction effect) has been ruled out.

We agree with this statement and have validated that controls were not significantly different before pooling data from different experiments. To clarify this point, we have added this sentence in the methods:
L. 699-701: “To justify pooling results from different experiments and ruling out any uncontrolled variable, we validated that controls were not significantly different.”

The authors could have discussed to which extent the choice of DENV type and strain (DENV2 NGC) could have influenced the results. All four DENV types share conserved functional RNA structures in their 3’UTR (except for DENV4), however the 3’UTR differs in sequence length and nucleotide composition between DENV types.

We have not confirmed the interactions with other DENV1, 3 and 4 3’UTR. However, from our previous experiments in human cells, the 3’UTR-interacting RBPs widely differ between the different serotypes (Liao et al, 2018. RNA). To clarify this point, we have now added this information:

L. 159-160: “As demonstrated previously in human [21], RBPs interacting with the 3’UTR of DENV2 may widely vary from RBPs interacting with the other DENV serotype 3’UTR.”

Minor comments:

Line 106: “interphase”, do you mean “interface”?

Thanks for point the mistake. We corrected for “interface”.

Line 210: Expressing a proportion as percentage of reduction is ambiguous because it could represent the absolute difference in percentage or the percentage of reduction, relative to the control. Please clarify.

We clarified this and the sentence now reads:
L. 229: “…silencing of *AeRan* and *AeExoRNase* reduced the percentage of infected mosquitoes by 23.5 and 29 points, respectively…”

Lines 249-250: Since the authors did not test HsPurG this statement could be softened.

The sentence now reads:
L. 269: “However, although we have not tested the function of HsPurG, our results suggest that the anti-viral function of AePur is not conserved in the human homologs.”

Line 396: “non-mediated”, do you mean “nonsense-mediated”?

The text was corrected accordingly.

Line 506: Is the blood meal titer stable for 1.5 hours?

We have not tested the blood meal titer after oral feeding in this study. Nonetheless, from previous experiments, the blood meal titer is twice lower after 1h. To account for this, we added this information in the methods:
L. 531: “The blood meal titer may have decreased after 1.5 h.”

Reviewer #2: None

Reviewer #3: 1. Among the fourteen proteins identified, some of them (such as AeGTPase) do not contain defined/known RNA binding domain. These proteins may associate with DENV2 3’UTR indirectly via other proteins and do not necessarily have a direct interaction with DENV2 3’UTR. Thus, it is more appropriate to use “association” than “binding” to describe the relation between these identified proteins and DENV2 3’UTR.

We thank the reviewer for pointing to this alternative hypothesis. To include this hypothesis, we have added the following sentence and changed the term “bind” for “associate” or “interact” throughout the text.

L. 157-159: “While most of the interacting RBPs have a RNA binding domain, these and the others could either directly bind to the 3’UTR or indirectly through a complex of proteins.”

2. Following sentences are a bit confusing and misleading. The authors might like to rephrase them.
• Line 141-142 “AeMaleless, an ATP-dependent helicase involved in dosage compensation [30]”. This sentence gives the impression that AeMaleless plays a role in dosage compensation. In fact, the role of AeMaleless has not been established. The role of Maleless in dosage compensation is reported in Drosophila melanogaster.
• Similarly, in Line 142-142 “AeSex-lethal, an RNA-binding protein also involved in dosage compensation” need to be rephrased.

We agree with the reviewer’s comment. The function was documented for the Drosophila homologs. We minimized our extrapolation from Drosophila’s studies by adding “**putatively**” involved in dosage compensation.

3. For each of fourteen candidate genes, ~0.2 ug of dsRNA was injected into mosquito to evaluate their potential function, while ~1.9 ug of dsRNA was delivered to address AeStaufen function in different tissues. The authors should explain the reason of using different amount of dsRNA in these experiments.

We have now clarified this point by adding the following sentence in the methods section:
L. 623-624: “We injected a higher quantity of dsRNA against AeStaufen to have an effective silencing in salivary glands.”

4. Line 201-202, “we controlled that survival was similar across the various silencing conditions”. How did the authors control the survival rate without affecting the knockdown efficiency? Similarly, in Line 270, “we controlled that blood feeding and survival rates were not affected by AeStaufen depletion…”. How was this experimented conducted without affecting knockdown efficiency? Line 298 “we controlled that the salivation rate was not affected by AeStaufen silencing…”. How was salivation rate experimentally controlled without affecting knockdown efficiency? The authors need to add the methodology used for these experiments.

We are sorry for the lack of clarity. Our idea was to make sure that the different gene silencing did not influence survival, blood feeding and salivation in order to avoid any bias toward selection of either lower or higher infected mosquitoes. With this in mind, we probably misused the term “control” as we did not modify silencing to regulate survival, blood feeding and salivation.
We now removed the term “control” and clarified that we measured survival, blood feeding and salivation rates in the different silencing conditions to evaluate the impact of gene silencing on these parameters (L. 219-221; L. 291-292; L. 320).

5. For the RIP experiment, anti-human PurB antibody is used to pull down AePur, it’d be great if the authors could conduct dsRNA knockdown experiment in cells to verify the specificity of this antibody toward AePur.

To answer the reviewer’s question, we confirmed the specificity of the anti-human PurB for AePur in mosquitoes. We now present in Fig S1 (L. 175) a WB of mosquitoes injected with dsRNA against AePur as compared to mosquitoes injected with control dsRNA. Supporting the specificity of the Pur antibody, the band detected with the anti-human PurB is decreased upon silencing.

6. There is a discrepancy of DENV2 titer used for AePur RIP experiment. It is mentioned as 10^	6 PFU/mL for AePer RIP (line 502-505), but it is stated as 10^7 PFU/mL. The authors need to correct one mistake here.

We used two different blood viral titers for AePur and AeStaufen. This was clearly mentioned in the methods. However, to clarify our methods, we added the blood titer that we used in the results section at each relevant position.

PLOS authors have the option to publish the peer review history of their article ([what does this mean?](https://journals.plos.org/plospathogens/s/editorial-and-peer-review-process#loc-peer-review-history)). If published, this will include your full peer review and any attached files.


If you choose “no”, your identity will remain anonymous but your review may still be made public.


**Do you want your identity to be public for this peer review?** For information about this choice, including consent withdrawal, please see our [Privacy Policy](https://www.plos.org/privacy-policy).

Reviewer #1: **Yes:** Louis Lambrechts

Reviewer #2: No

Reviewer #3: No
